# 5G wavelength-division-multiplexing-based bidirectional optical wireless communication system with signal remodulation employing cascaded reflective semiconductor optical amplifiers

**DOI:** 10.1038/s44172-024-00232-1

**Published:** 2024-06-22

**Authors:** Hai-Han Lu, Chung-Yi Li, Xu-Hong Huang, Yu-Yao Bai, Wei-Wen Hsu, Yu-Chen Chung, Jia-Ming Lu, Kelper Okram

**Affiliations:** 1 Institute of Electro-Optical Engineering, National Taipei University of Technology, Taipei, 10608 Taiwan; 2 Department of Communication Engineering, National Taipei University, New Taipei City, 23730 Taiwan; 3 The School of Information Science and Engineering, Fujian University of Technology, Fujian, 350118 China

**Keywords:** Fibre optics and optical communications, Microwave photonics

## Abstract

Compared with previous generations, fifth-generation communications can provide faster download and upload speeds and support a greater number of connected devices. Integrating fifth-generation signals with optical wireless communication systems provides promising ways to afford higher transmission rates and faster wireless connectivity. Here we report a fifth-generation wavelength-division-multiplexing-based bidirectional optical wireless communication system with signal remodulation employing cascaded reflective semiconductor optical amplifiers to effectively remove the downstream data for uplink transmission. It shows a fifth-generation wavelength-division-multiplexing-based bidirectional optical wireless communication system using four wavelengths for communication. The uplink performance is substantially enhanced by using two reflective semiconductor optical amplifiers to remove the downstream data. The system achieves an aggregate transmission rate of 36.4 Gbit/s for both downlink and uplink transmissions over a 100-m optical wireless link. This demonstrated fifth-generation wavelength-division-multiplexing-based bidirectional optical wireless communication system employing cascaded reflective semiconductor optical amplifiers holds great potential for enhancing fifth-generation advanced communication capabilities.

## Abstract (web summary)

Lu et al. demonstrated a bidirectional optical wireless communication system for 5G communications using wavelength-division multiplexing and cascaded reflective semiconductor optical amplifiers. The system achieves an aggregate rate of 36.4 Gb/s over a 100-m optical wireless link, enhancing 5G communication capabilities.

## Introduction

**Fig. 1:**
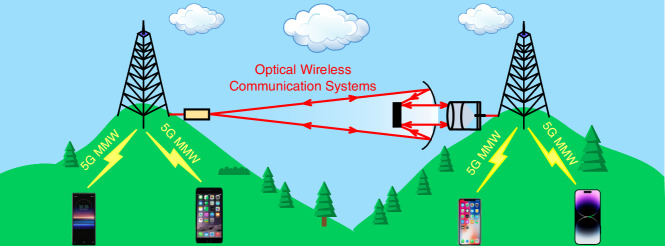
The integration of 5G (5G, fifth-generation) signals with OWC (OWC, optical wireless communication) systems. The integration of 5G signals with OWC systems offers promising avenues for providing high transmission rates and meeting the growing demand for faster and more reliable wireless connectivity.

Fifth-generation (5G) communications deliver a substantial boost in transmission rates due to the combination of expanded bandwidth and sophisticated communication techniques^1–4^. It enables new applications such as mixed reality, cloud gaming, and real-time IoT applications. With the rapid development of optical wireless communication (OWC) systems, they have gained attention due to their potential for providing high-speed and high-capacity optical communications, especially for scenarios where radio frequency communications are challenging^5,6^. The integration of 5G signals with OWC systems (as illustrated in Fig. 1) therefore offers promising avenues for providing high transmission rates and meeting the growing demand for faster and more reliable wireless connectivity. Former research presented the feasibility of building an actively controllable beam steering OWC system employing an integrated optical phased array^7^. However, it did not directly connect 5G signal through optical wireless links. 5G signals through optical wireless links are important for the integration of 5G signals with OWC systems. One of the characteristics of the 5G OWC system is that it is directly related to 5G communications. In actual scenarios, a 5G OWC system should be developed instead of an OWC system that is not connected to 5G communications. Besides, such an actively controllable beam steering OWC system is a unidirectional OWC system, not a bidirectional OWC system. A bidirectional OWC system allows simultaneous transmission in both downlink and uplink transmissions. It enables free space reuse and better spectrum utilization, leading to higher transmission rates and capacities. Furthermore, constructing a bidirectional OWC system with phase modulation and remotely injection-locked distributed feedback laser diode (LD) was shown to be practicable^8^. Nevertheless, it presents challenges in terms of converting phase modulation to intensity modulation using remotely injection-locked distributed feedback LD. In addition, four-level pulse amplitude modulation and non-return-to-zero signals are used and transmitted in this bidirectional OWC system. However, the third-generation partnership project (3GPP) specifications do not define these signal types. In contrast, the 5G signal defined by 3GPP specifications uses an orthogonal frequency-division multiplexing (OFDM) signal for both downlink and uplink transmissions^9^. Obviously, there is room for improvement in the 5G signal transmission supported by 3GPP specifications. Moreover, a 40-Gbit/s downlink signal transport using an OFDM signal with single sideband modulation, and a 10-Gbit/s uplink signal transport employing a reflective semiconductor optical amplifier (RSOA) for remodulation were realized^10^. However, it has not aligned with 5G signal transmission. Bidirectional lightwave transport systems should be developed to align with 5G communications. In this demonstration, a 5G wavelength-division-multiplexing (WDM)-based bidirectional OWC system with signal remodulation employing cascaded RSOAs to effectively remove the downstream data for uplink transmission is practically implemented. It shows a 5G WDM-based bidirectional OWC system using four optical wavelengths and two RSOAs as a demonstration. For downlink transmission, each of the four optical wavelengths is used to deliver a 9.1-Gbit/s/28-GHz millimeter-wave (MMW) signal using 16-quadrature amplitude modulation (QAM)-OFDM modulation. The downstream modulated data on the four optical wavelengths is effectually erased by two RSOAs, and these wavelengths are then reused for upstream optical carriers. The uplink performance is substantially enhanced by utilizing two RSOAs to remove the downstream data. The four upstream optical wavelengths are modulated by an MZM with 9.1-Gbit/s/24-GHz MMW signal using 16-QAM-OFDM modulation. This 5G WDM-based bidirectional OWC system achieves an aggregate transmission rate of 36.4 Gbit/s (9.1 Gbit/s × 4) for both downstream and upstream data. Through 100-m optical wireless link, good bit error rates (BERs) (<3.8 × 10^−3^ forward error correction (FEC) limit) and error vector magnitudes (EVMs) (<12.5% 3GPP limit) performance^11,12^, clear constellation diagrams, and flat electrical spectra are attained for downlink/uplink transmissions.

5G WDM-based bidirectional OWC systems are expected to use 4 laser sources for the upstream. However, this process increases the complexity to 5G WDM-based bidirectional OWC systems. For an actual implementation of 5G WDM-based bidirectional OWC systems, the promotion of simple upstream light source is vital. Employing two RSOAs to remove the downstream data offers an attractive solution because it does not require the use of four lasers with chosen wavelengths for uplink transmission. By utilizing two RSOAs instead of four lasers with selected wavelengths, the complexity associated with wavelength selection can be avoided. In addition, if four inventory lasers with different wavelengths but not selected wavelengths are used for uplink transmission, the WDM demultiplexer (DEMUX) at the upstream receiving site cannot accurately demultiplex the upstream wavelength. Inaccurate demultiplexing due to the use of lasers with non-selected wavelengths will degrade uplink transmission performance. Signal remodulation can be achieved through different devices such as Fabry-Perot (FP) LD^13^, RSOA^14,15^, and electro-absorption modulator^16^. Due to the limited bandwidth of FP LD and RSOA, it is challenging for FP LD and RSOA to provide high-speed data streams for uplink transmission. For the electro-absorption modulator, the uplink transmission performance based on FP LD and RSOA is better than that based on the electro-absorption modulator^16^. In our proposed bidirectional OWC system, the four upstream wavelengths are modulated by a Mach-Zehnder modulator (MZM). The bandwidth of MZM is greatly higher than that of FP LD and RSOA, which can provide higher data streams for uplink transmission. Besides, previous works on the signal remodulation of optical MMW signals have been reported^17,18^. However, complex vertical cavity surface emitting laser-based phase modulation to intensity modulation converter and sophisticated centralized light source for uplink transmission are required. Furthermore, since only one optical carrier is reused in the uplink, the uplink transmission rate will be much lower than that associated with operation using multiple optical carriers. Furthermore, downstream data erasure can be achieved by using an RSOA with low saturation input power. When operating an RSOA with low saturation input power, increasing the RSOA input power can further erase the downstream data. However, owing to the limited slope of the output power-input power curve, this approach may encounter challenges related to incomplete erasure, resulting in residual downstream data.

Using two RSOAs to completely erase the downstream data and then using MZM to modulate four optical carriers is worthwhile as they avoid the requirement for multiple lasers with selected wavelengths and the bandwidth limitation of using FP LD, RSOA, and electro-absorption modulator. Furthermore, they do not require complex vertical-cavity surface-emitting laser-based phase modulation to intensity modulation converter and sophisticated centralized light source. Additionally, they also avoid the constraint of selecting an RSOA with low saturation input power. By using RSOAs to erase the downstream data, the same optical carriers can be reused for uplink transmission, thereby optimizing spectral efficiency. Moreover, RSOAs offer a compact and straightforward solution for implementing bidirectional OWC systems. This eliminates the need for separate optical devices or complex signal processing techniques, simplifying system architecture and reducing implementation complexity. Additionally, the use of RSOAs provides greater flexibility in system deployment, as it eliminates the need for dedicated wavelength management. Using RSOAs in bidirectional OWC systems offers substantial advantages in spectral efficiency, simplicity, and flexibility in deployment, meeting the demands of efficient 5G WDM-based bidirectional OWC systems. The deployment of 5G WDM-based bidirectional OWC system using cascaded RSOAs for signal remodulation is an important step in realizing 5G communications through bidirectional OWC systems. It acts as a reference model for future development and a successful model for next-generation communications.

## Results and Discussion

### Downlink/uplink BERs/EVMs and associated constellation diagrams

**Fig. 2:**
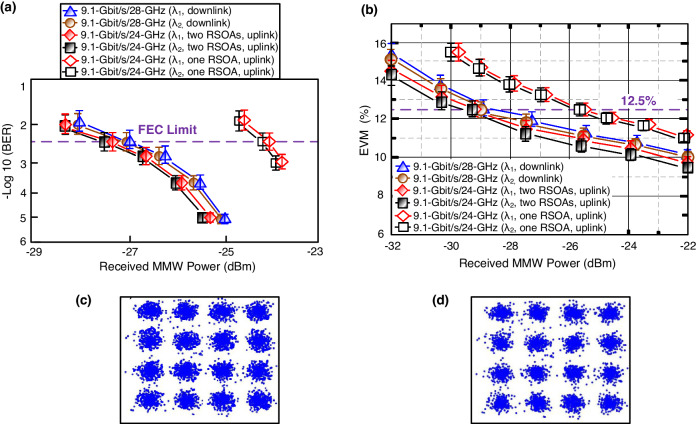
Downlink/uplink BERs (BERs, bit error rates)/EVMs (EVMs, error vector magnitudes) and associated constellation diagrams. **a** BERs and **b** EVMs at different received MMW (MMW, millimeter-wave) powers over 100-m optical wireless link, at wavelengths of λ_1_ and λ_2_ (downlink/uplink). Blue triangle/line indicates 9.1-Gbit/s/28-GHz signal (λ_1_, 1549.3 nm; downlink), brown circle/line indicates 9.1-Gbit/s/28-GHz signal (λ_2_, 1550.9 nm; downlink), red rhombus/line indicates 9.1-Gbit/s/24-GHz signal (λ_1_, two RSOAs, uplink), black square/line indicates 9.1-Gbit/s/24-GHz signal (λ_2_, two RSOAs, uplink), red hollow rhombus/line indicates 9.1-Gbit/s/24-GHz signal (λ_1_, one RSOA, uplink), and black hollow square/line indicates 9.1-Gbit/s/24-GHz signal (λ_2_, one RSOA, uplink). The error bars represent the standard deviations of the measured data from three experimental trials. The associated constellation diagrams of (**c**) 9.1-Gbit/s/28-GHz and **d** 9.1-Gbit/s/24-GHz 16-QAM-OFDM (16-QAM-OFDM, 16-quadrature amplitude modulation-orthogonal frequency-division multiplexing) signals over 100-m optical wireless link, at wavelength of λ_1_ (downlink/uplink) with a BER of 3.8 × 10^−3^ and 2.6 × 10^−3^.

The downlink/uplink BERs under different received MMW powers over a 100-m optical wireless link are exhibited in Fig. 2a. To demonstrate the remodulation, two wavelengths of λ_1_ (1549.3 nm) and λ_2_ (1550.9 nm) are chosen for downlink and uplink BER performance evaluation. It is to be seen that the BER performance is nearly the same for λ_1_ and λ_2_ in the downlink and uplink transmissions. Results indicate that the choice of wavelength has a minimal impact on the downlink/uplink BER performance. Moreover, due to the utilization of a 30-GHz photodiode (PD) in the experiment, 28-GHz signals require a higher received MMW power compared to 24-GHz signals for a similar BER of the system. For downlink OFDM signal transmission, a 3.8 × 10^−3^ (FEC limit) BER is attained at received MMW powers of −26.9 (λ_1_, 1549.3 nm) and −27 (λ_2_, 1550.9 nm) dBm, as measured by a spectrum analyzer. For uplink OFDM signal transmission (two RSOAs), a 3.8 × 10^−3^ BER is acquired at received MMW powers of −27.2 (λ_1_) and −27.3 (λ_2_) dBm. To have more correlation with the number of RSOA and uplink BER performance, we remove one RSOA to evaluate the uplink BERs over 100-m optical wireless link. For uplink OFDM signal transmission (one RSOA), a 3.8×10^−3^ BER is acquired at received MMW powers of −23.9 (λ_1_) and −24.1 (λ_2_) dBm. At 3.8 × 10^−3^ BER, power penalty improvements of 3.3 dB (λ_1_) and 3.2 dB (λ_2_) are observed when using two RSOAs. The use of two RSOAs contributes to the further erasure of downstream modulated data. There is no residual downstream modulated data and the uplink BER is substantially improved. Furthermore, Fig. 2b shows the EVMs at wavelengths of λ_1_ and λ_2_ (downlink/uplink) and at different received MMW powers. Through a 100-m optical wireless link, the EVMs of downlink/uplink 16-QAM-OFDM signals remain below the 12.5% 3GPP limit when the received MMW powers are higher than −28.9 (λ_1_), −29.1 (λ_2_), −29.3 (λ_1_, two RSOAs, uplink), and −29.4 (λ_2_, two RSOAs, uplink) dBm, respectively. The downlink 9.1-Gbit/s/28-GHz 16-QAM-OFDM signal has a slightly lower receiver sensitivity compared to the uplink 9.1-Gbit/s/24-GHz 16-QAM-OFDM signal. Moreover, since EVM is related to the carrier frequency and peak-to-average power ratio, a 9.1-Gbit/s/24-GHz 16-QAM-OFDM signal has a lower peak-to-average power ratio, contributing to a lower EVM at the same received power^19,20^. To verify the relationship between the number of RSOA and uplink EVM performance, we change two RSOAs to one RSOA to evaluate the EVMs. For the uplink signal using 16-QAM-OFDM modulation (one RSOA), a 12.5% EVM is obtained at received MMW powers of −25.5 (λ_1_) and −25.7 (λ_2_) dBm. At 12.5% EVM, power penalty degradations of 3.8 dB (λ_1_) and 3.7 dB (λ_2_) exist when using one RSOA. When using one RSOA, the downstream modulated data is incompletely erased, leading to interference to degrade the uplink EVM performance. As for the constellation diagrams, Figs. 2c and d show the connected constellation diagrams of 9.1-Gbit/s/28-GHz and 9.1-Gbit/s/24-GHz 16-QAM-OFDM signals at a wavelength of λ_1_ (downlink/uplink), over 100-m optical wireless link and at −26.9-dBm received MMW power. Clearly, each downlink/uplink 16-QAM-OFDM signal has a distinct constellation diagram with BER of 3.8 × 10^−3^ (Fig. 2c) and 2.6 × 10^−3^ (Fig. 2d). Low BERs, low EVMs, and clear constellation diagrams support the feasibility and utility of using 5G MMW signals over a bidirectional OWC with two RSOAs.

### Comparisons for constellation diagrams with scenarios involving no RSOA, one RSOA, and two RSOAs

**Fig. 3:**
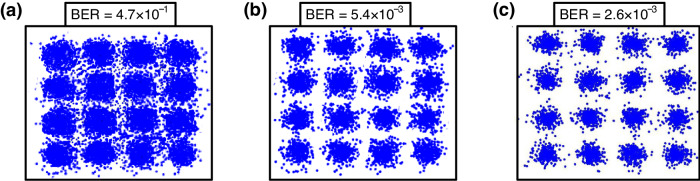
Comparisons for constellation diagrams with scenarios involving no RSOA (RSOA, reflective semiconductor optical amplifier), one RSOA, and two RSOAs. The related constellation diagrams of 9.1-Gbit/s/24-GHz 16-QAM-OFDM (16-QAM-OFDM, 16-quadrature amplitude modulation-orthogonal frequency-division multiplexing) signal at wavelength of λ_1_ in the scenarios with **a** no RSOA, **b** one RSOA, and **c** two RSOAs, over 100-m optical wireless link and at −27.2-dBm received MMW (MMW, millimeter-wave) power.

To clarify the improvement atained by employing two RSOAs, comparisons for constellation diagrams with scenarios involving no RSOA, one RSOA, and two RSOAs are presented. Figures 3a–c show the related constellation diagrams of 9.1-Gbit/s/24-GHz 16-QAM-OFDM signal at wavelength of λ_1_ (uplink) through 100-m optical wireless link and at −27.2-dBm received MMW power, in the scenarios with no RSOA, one RSOA, and two RSOAs. In the scenario with no RSOA, the constellation diagram shows a blurred pattern with 4.7×10^−1^ BER (Fig. 3a). In the scenario with one RSOA, the constellation diagram presents a somewhat blurred pattern with 5.4×10^−3^ BER (Fig. 3b). In the scenario with two RSOAs, however, a clear and distinct constellation diagram with 2.6×10^−3^ BER (Fig. 3c) is attained. In the scenario with no RSOA, the downstream modulated data is not erased and this can lead to the simultaneous modulation of downstream and upstream data on the same optical carrier. This situation brings on strong interference that degrades uplink performance and results in a blurred constellation diagram. In the scenario with one RSOA, the downstream modulated data is incompletely suppressed and this can lead to partial downstream data and all upstream data modulating the same optical carrier. This situation can cause interference, leading to reduced uplink performance and somewhat blurred constellation diagrams. As for the scenario with two RSOAs, the downstream modulated data is virtually eliminated, ensuring that only upstream modulated data exists on the optical carrier. This situation will not cause interference from the downstream modulated data therefore improving the uplink performance and making the constellation diagrams clearly visible. The clear and distinct constellation diagrams show that this proposed bidirectional OWC systems with signal remodulation employing cascaded RSOAs is capable of transmitting 5G signals at MMW frequencies.

### The electrical spectra of 9.1-Gbit/s/28-GHz and 9.1-Gbit/s/24-GHz 16-QAM-OFDM signals, in the scenarios of using one RSOA and two RSOAs

**Fig. 4:**
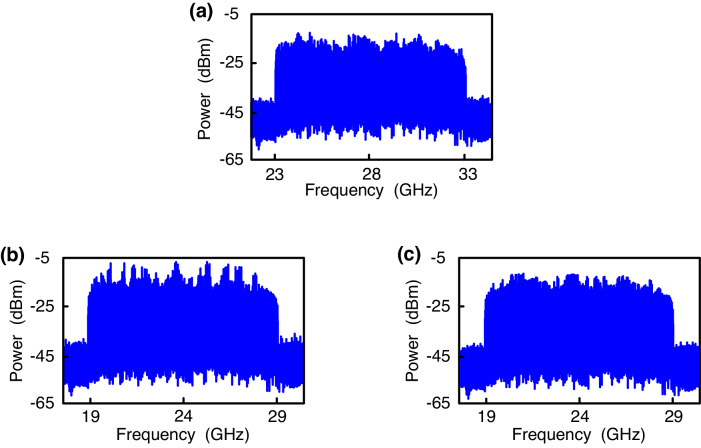
The electrical spectra of 9.1-Gbit/s/28-GHz and 9.1-Gbit/s/24-GHz 16-QAM-OFDM (16-QAM-OFDM, 16-quadrature amplitude modulation-orthogonal frequency-division multiplexing) signals. **a** The electrical spectrum of 9.1-Gbit/s/28-GHz 16-QAM-OFDM signal at λ_1_ wavelength, through 100-m optical wireless link and at −26.9-dBm received MMW (MMW, millimeter-wave) power. The electrical spectra of 9.1-Gbit/s/24-GHz 16-QAM-OFDM signal at λ_1_ wavelength in the scenarios of using (**b**) one RSOA (RSOA, reflective semiconductor optical amplifier) and **c** two RSOAs, through 100-m optical wireless link and at −27.2-dBm received MMW power.

Figure 4a, b (one RSOA), and Fig. 4 (two RSOAs) depict the electrical spectra of 9.1-Gbit/s/28-GHz (λ_1_, downlink) and 9.1-Gbit/s/24-GHz (λ_1_, uplink) 16-QAM-OFDM signals through 100 m optical wireless transmission and at −26.9 and −27.2-dBm received MMW powers. The electrical spectrum of the downlink OFDM signal (Fig. 4a) has an acceptable amplitude fluctuation within ±3.5 dB. Since higher frequency signals experience higher propagation losses, the downlink OFDM signal has slightly higher powers in 23-31 GHz frequencies compared to the 31–33 GHz frequencies^21,22^. In the scenario when only one RSOA is employed, the electrical spectrum of the uplink OFDM signal (Fig. 4b) exhibits a large amplitude fluctuation within ±6.8 dB. Such a large amplitude fluctuation is attributed to the incomplete suppression of downstream modulated data and consequently results in worse signal quality and large amplitude fluctuations. In the scenario of using two RSOAs, the electrical spectrum of the uplink OFDM signal (Fig. 4c) shows small amplitude fluctuations within ±3.1 dB. This shows that the downstream modulated data is virtually suppressed. Using two RSOAs mitigates interference from downstream modulated data, resulting in better uplink performance and smaller amplitude fluctuations.

### The subcarrier EVMs of the 9.1-Gbit/s/28-GHz (downlink) and 9.1-Gbit/s/24-GHz (uplink) 16-QAM-OFDM signals

**Fig. 5:**
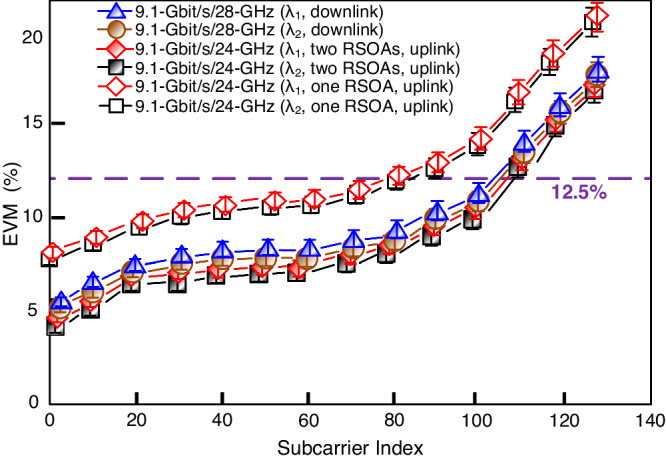
The subcarrier EVMs (EVMs, error vector magnitudes) of the 9.1-Gbit/s/28-GHz (downlink) and 9.1-Gbit/s/24-GHz (uplink) 16-QAM-OFDM (16-QAM-OFDM, 16-quadrature amplitude modulation-orthogonal frequency-division multiplexing) signals. The subcarrier EVMs of the 9.1-Gbit/s/28-GHz (downlink) and 9.1-Gbit/s/24-GHz (uplink) 16-QAM-OFDM signals at different subcarrier indices, in the scenarios of using one RSOA (RSOA, reflective semiconductor optical amplifier) and two RSOAs. Blue triangle/line indicates 9.1-Gbit/s/28-GHz signal (λ_1_, 1549.3 nm; downlink), brown circle/line indicates 9.1-Gbit/s/28-GHz signal (λ_2_, 1550.9 nm; downlink), red rhombus/line indicates 9.1-Gbit/s/24-GHz signal (λ_1_, two RSOAs, uplink), black square/line indicates 9.1-Gbit/s/24-GHz signal (λ_2_, two RSOAs, uplink), red hollow rhombus/line indicates 9.1-Gbit/s/24-GHz signal (λ_1_, one RSOA, uplink), and black hollow square/line indicates 9.1-Gbit/s/24-GHz signal (λ_2_, one RSOA, uplink). The error bars represent the standard deviations of the measured data from three experimental trials.

Figure 5 exhibits the subcarrier EVMs of the 9.1-Gbit/s/28-GHz (downlink) and 9.1-Gbit/s/24-GHz (uplink) 16-QAM-OFDM signals at different subcarrier indices, in the scenarios of using one RSOA and two RSOAs. Similarly, λ_1_ (1549.3 nm) and λ_2_ (1550.9 nm) are picked for downlink and uplink subcarrier EVMs evaluation. After a 100-m optical wireless link, the EVMs of downlink OFDM signals remain below the 3GPP limit of 12.5% for subcarrier indices smaller than 104 (λ_1_) and 105 (λ_2_). Apparently, as the subcarrier index increases, the EVM also increases. The average EVMs for λ_1_ and λ_2_ are around 9.4% and 9.1%, respectively, both below the 12.5% 3GPP limit. It is also observed that through a 100-m optical wireless link and in the scenario of using two RSOAs, the EVMs of the uplink OFDM signals are below the 3GPP limit when the subcarrier indices are smaller than 106 (λ_1_) and 107 (λ_2_). The average EVMs for λ_1_ and λ_2_ are around 8.6% and 8.4%, respectively, both below the 12.5% 3GPP limit. In addition, with only one RSOA, the EVMs of uplink OFDM signals remain below the 3GPP requirement when the subcarrier indices are less than 82 (λ_1_) and 83 (λ_2_). Note that the subcarrier indices of 82 (λ_1_) and 83 (λ_2_) (using one RSOA) are much smaller than the corresponding values of 106 (λ_1_) and 107 (λ_2_) (using two RSOAs). The average EVMs are approximately 12.8% (λ_3_) and 12.6% (λ_4_), which are above the 3GPP limit of 12.5%. The high average EVM is caused by using one RSOA to incompletely erase the downstream modulated data. Two RSOAs can further remove the downstream data. Therefore, the uplink EVMs can be further reduced by using two RSOAs to effectively remove the downstream data. The average downlink/uplink EVMs below the 12.5% 3GPP limit show the practicability of building 5G WDM-based bidirectional OWC systems employing cascaded RSOAs to efficiently remove the downstream data.

Using QAM-OFDM modulation with bit-loading technique is a powerful method to increase aggregate transmission rates^23–26^. Subcarriers with higher signal-to-noise ratio support higher-QAM-OFDM modulation (e.g., 64-QAM-OFDM or 128-QAM-OFDM). Conversely, subcarriers with lower signal-to-noise ratio support lower-QAM-OFDM modulation (e.g., 4-QAM-OFDM or 16-QAM-OFDM). There is an inverse relationship between signal-to-noise ratio and the square of EVM, subcarriers with lower EVM can adopt higher-QAM-OFDM modulation, while subcarriers with higher EVM can adopt lower-QAM-OFDM modulation^27–29^. This dynamic allocation of 2^n^-QAM-OFDM modulation optimizes the use of available resources and results in higher aggregate transmission rates. By adapting the modulation to the specific quality of each subcarrier, the system maximizes its overall data transmission capacity.

## Methods

### 5G WDM-based bidirectional OWC systems with cascaded RSOAs

**Fig. 6:**
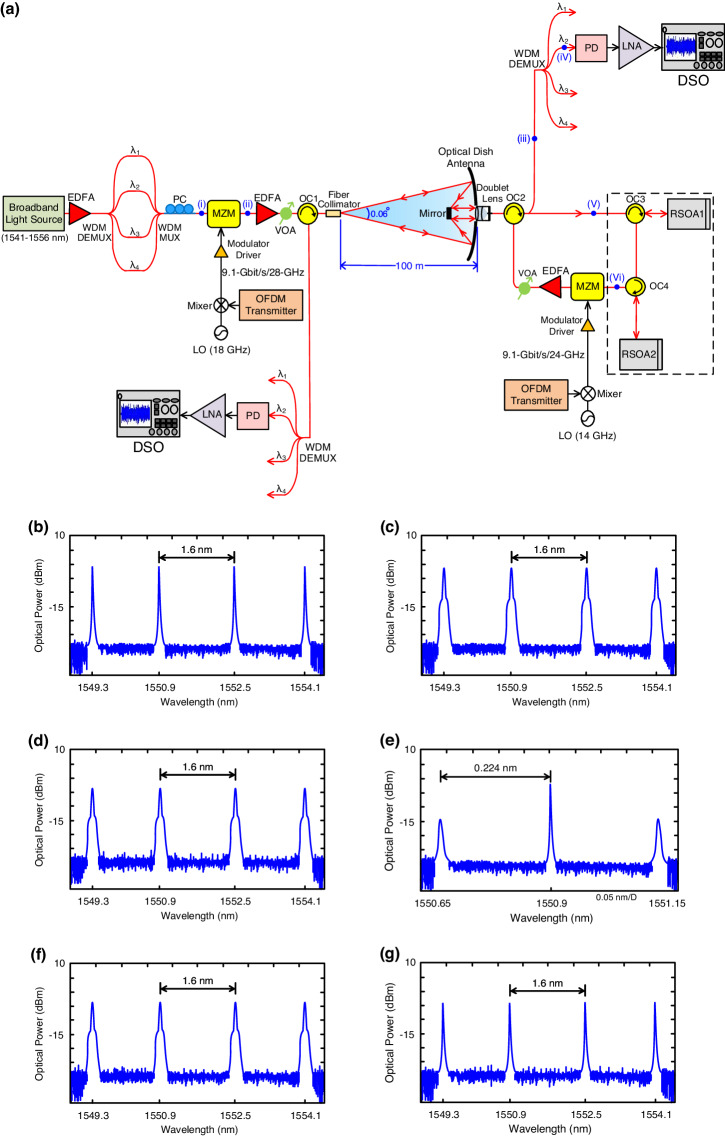
5G (5G, fifth-generation) WDM (WDM, wavelength-division-multiplexing)-based bidirectional OWC (OWC, optical wireless communication) systems with cascaded RSOAs (RSOAs, reflective semiconductor optical amplifiers). **a** Configuration of 5G WDM-based bidirectional OWC systems with signal remodulation employing cascaded RSOAs. The optical spectra (**b**) before the MZM at the transmitting site, **c** after the MZM at the transmitting site, **d** before the WDM DEMUX at the receiving site, **e** after the WDM DEMUX at the receiving site, **f** before two RSOAs, and (**g**) after two RSOAs. EDFA erbium-doped fiber amplifier, DEMUX demultiplexer, PC polarization controller, MZM Mach-Zehnder modulator, OFDM orthogonal frequency-division multiplexing, LO local oscillator, VOA variable optical attenuator, OC optical circulator, MUX multiplexer, PD photodiode, LNA low noise amplifier, DSO digital sampling oscilloscope.

Figure 6a depicts the configuration of the 5G WDM-based bidirectional OWC systems with signal remodulation employing cascaded RSOAs. An actual point-to-point system is built rather than a simulated one. The output of the broadband light source, with 15 nm bandwidth (1541–1556 nm) and ±1.4 dB flatness, is boosted by an erbium-doped fiber amplifier (EDFA) and efficiently separated into four wavelengths by a 1×4 WDM DEMUX. The four wavelengths of λ_1_ (1549.3 nm), λ_2_ (1550.9 nm), λ_3_ (1552.5 nm), and λ_4_ (1554.1 nm) are multiplexed by a 4 × 1 WDM MUX, and then supplied to an MZM via a polarization controller. Both the WDM MUX and DEMUX feature 1.6 nm wavelength spacing, 0.6 nm channel passband width, and >30 dB channel isolation. The 9.1-Gbit/s/10-GHz 16-QAM-OFDM signal generated by the OFDM transmitter is upconverted to a 9.1-Gbit/s/28-GHz signal using a mixer with an 18 GHz local oscillator signal. The upconverted signal then drives an MZM through a modulator driver. The optical spectra before and after the MZM are shown in Figs. 6b, c (insert (i) and (ii) of Fig. 6a). Four modulated optical wavelengths travel through an EDFA with a flat amplifier gain of ±1.3 dB over 30 nm (1530–1560 nm) for WDM applications. A variable optical attenuator is positioned at the beginning of the optical wireless link so that the optical power emitted into free space can be optimized to achieve the best link performance. Via two optical circulators (OC1 and OC2), the optical signals are delivered through a 100-m optical wireless link using a fiber collimator with 0.06° divergence angle and 1050-1620 nm wavelength range at the transmitting site and an optical dish antenna with a doublet lens at the receiving site. An optical dish antenna is a dish antenna with 90 cm diameter and high reflectivity (>99%) mirrors that firstly focus the laser light to a mirror at the focal point and next reflect the laser light to the fiber ferrule of the doublet lens. The diameter of the laser light on the optical dish antenna is 20 cm. Since the system is based on point-to-point OWC link and the laser travels through a 100-m optical wireless link, fully steering the laser beam to the input of the fiber ferrule remains a challenge^30,31^. An optical equipment should be arranged to decrease the laser beam size to fully steer the laser beam to the input of the fiber ferrule. A Doublet lens at the receiving site is an optical equipment that steers the laser beam to the input of the fiber ferrule. After circulation by the OC2, the optical signal with four wavelengths is split into two parts utilizing an optical splitter. One part of the optical signal is demultiplexed by a 1×4 WDM DEMUX, which has the same characteristics as the WDM DEMUX at the transmitting site. The optical spectra before and after the WDM DEMUX are presented in Figs. 6d, e (insert (iii) and (iv) of Fig. 6a). Due to a large channel passband width of 0.6 nm, the modulation sidebands can be detected by the WDM DEMUX. The demultiplexed wavelength with downlink OFDM signal is received by a 30-GHz PD, amplified by a low noise amplifier with frequencies of 2-30 GHz and a noise figure of 2.4 dB, and transmitted to a digital sampling oscilloscope for downlink performance estimation. Another part of the optical signal is injected into two RSOAs (RSOA1 and RSOA2) via the OC3 and the OC4 to virtually erase the downstream modulated data and reproduce four pure optical carriers for uplink transmission. The optical spectra before and after two RSOAs are exhibited in Figs. 6f, g (insert (v) and (vi) of Fig. 6a). RSOA1 has a bandwidth of 3.6 GHz and a seeding power range of −22 to −10 dBm. The optical properties of RSOA2 are the same as RSOA1. The function of RSOA1 is to erase the downstream data from incoming optical signals. The optical carriers with residual data are subsequently injected into RSOA2, which further erases the residual data. The downstream modulated data is effectively suppressed through unsaturated RSOA1 and gain-saturated RSOA2^32^. RSOA1 operates at 60 mA bias current and −18 dBm seeding power, and RSOA2 operates at 100 mA bias current and −12 dBm seeding power.

For uplink transmission, a 9.1-Gbit/s/10-GHz 16-QAM-OFDM signal is upconverted to a 9.1-Gbit/s/24-GHz signal using a mixer with a 14 GHz local oscillator signal. Then, the upconverted signal drives an MZM through a modulator driver. After amplification by an EDFA, a variable optical attenuator optimally controls the optical powers. Through routing by two optical circulators (OC2 and OC1), the optical signal with four wavelengths is transmitted wirelessly through a 100-m optical wireless link using a doublet lens with an optical dish antenna. At the receiving site, a fiber collimator is used to collect the transmitted optical signal. The received optical signal is demultiplexed by a 1×4 WDM DEMUX. The demultiplexed wavelength with uplink OFDM signal is received by a 30-GHz PD and amplified by a low noise amplifier with frequencies of 2-30 GHz. The enhanced electrical signal is next fed into a digital storage oscilloscope for uplink performance analysis.

Additionally, a table (Table 1) outlines the chief parameters of the experiment such as 16-QAM-OFDM, signal format, fiber collimator, optical dish antenna, doublet lens, PD, low noise amplifier, and RSOA1/RSOA2.

**Table 1:** The chief parameters of the experiment

Chief Parameter	16-QAM-OFDM	Signal Format	Fibre Collimator	Optical Dish Antenna
Value	9.1 Gbit/s •128 subcarriers 120 data subcarriers 8 pilot subcarriers •512 FFT size •16 CP samples •10 G samples per second	Downlink •9.1-Gbit/s/28-GHz Uplink •9.1-Gbit/s/24-GHz	•0.06**°** divergence angle •1050-1620 nm	•90 cm diameter •high reflectivity ( >99%) mirrors
**Chief Parameter**	**Doublet Lens**	**PD**	**LNA**	**RSOA1/RSOA2**
Value	•concave lens + convex lens •50.8 nm diameter •150 mm focal length	Downlink/Uplink •30 GHz bandwidth	Downlink/Uplink •2–30 GHz frequencies •2.4 dB noise figure	•3.6 GHz bandwidth •−22 **～** −10 dBm seeding power Range •RSOA1: 60 mA bias current •−18 dBm seeding power •RSOA2: 100 mA bias current −12 dBm seeding power

The chief parameters of the experiment such as 16-QAM-OFDM (16-QAM-OFDM, 16-quadrature amplitude modulation-orthogonal frequency-division multiplexing), signal format, fiber collimator, optical dish antenna, doublet lens, PD (PD, photodiode), LNA (LNA, low noise amplifier), and RSOA1/RSOA2 (RSOA, reflective semiconductor optical amplifier).

### 16-QAM-OFDM modulation/demodulation and data rate calculation

The OFDM modulation comprises serial-to-parallel conversion, QAM symbol mapping, inverse fast Fourier transform, parallel-to-serial, cyclic prefix insertion, and digital-to-analog conversion. The number of data subcarriers, pilot subcarriers, CP samples, and the length of FFT size is set to 120, 8, 16, and 512, respectively, in an OFDM symbol. The pilot subcarriers are essential for tracking and compensating for phase shifts introduced by the channel. They help maintain the integrity of the modulated data carried by the data subcarriers^33,34^. 512 samples exist in the data set. 16 CP samples are inserted into the data set. Therefore, the entire number of samples becomes 528. In each IFFT operation, 480 (120 carriers × 4 bits) informative data bits are transmitted. For a set of 512 data samples, there is 480/512 = 0.9375 bit/sample. To add the 16 samples CP to the 512 samples, an entire of 528 (512 + 16) samples are obtained. For a set of 528 data samples, there is 480/528 = 0.91 bit/sample. Digital-to-analog conversion transports samples at 10 GSa/s rate. The data rate can be counted as 0.91 × 10 = 9.1 Gbit/s. Thus, the data rate acquired by transporting a given set of samples is 9.1 Gbit/s. For downlink transmission, each optical wavelength therefore carries a 9.1-Gbit/s/28-GHz 16-QAM-OFDM signal. For uplink transmission, each optical wavelength therefore carries a 9.1-Gbit/s/24-GHz 16-QAM-OFDM signal.

### Calculation for the divergence angle of the fiber collimator

A 100-m optical wireless link comprises a fiber collimator with a divergence angle of *θ* (degree) at the transmitting site and an optical dish antenna with a doublet lens at the receiving site. Through a 100-m optical wireless link, the diameter of the laser light on the optical dish antenna (*D*) is 20 cm and can be expressed as

1 $$D=2.100 \, \left(m\right)\cdot \left(\theta \cdot \frac{\pi }{180}\right)=0.2(m)$$

From Eq. (1), the divergence angle (*θ*) of the fiber collimator can be derived as

2 $$\theta =\frac{0.2}{2\times 100}\cdot \frac{180}{\pi }=0.06$$

Results show that a 100-m optical wireless link can be successfully achieved by using a fiber collimator with a divergence angle of 0.06° at the transmitting site and an optical dish antenna with a laser diameter of 20 cm at the receiving site.

In addition, to fit an *l*-m free-space optical link with a set of doublet lenses into a 100-m optical wireless link with a fiber collimator and an optical dish antenna, through an *l*-m free-space optical link the laser diameter (*d*) should be equal to or less than 0.2 m (200 mm). For a doublet lens with 75 mm diameter and 150 mm focal length, the diameter of laser light at the transmitting site (*d*_T_) is derived as 45 mm [2 × 150 (focal length) × 0.15 (fiber numerical aperture)]. The corresponding beam radius and the divergence angle of laser light are derived as 3.6 μm and 24 × 10^−6^ radians, respectively. With these conditions, we can obtain:

3 $$d=\sqrt{{{d}_{T}}^{2}+{\left(2\theta l\right)}^{2}}=\sqrt{{45}^{2}+{(0.048l)}^{2}}\le 200({mm})$$

*l* (maximum) is calculated as 4060 m, meaning that a 100-m optical wireless link using a fiber collimator at the transmitting site and an optical dish antenna at the receiving site is equivalent to a 4060-m free-space optical link utilizing a set of doublet lenses at the transmitting and receiving sites.

### Supplementary information

Description of Additional Supplementary Files

Supplementary Data 1

Supplementary Data 2

Supplementary Data 3

## Data Availability

The data in this manuscript are available from the corresponding author upon reasonable request. The source data for Fig. 2a, b, and Fig. 5 are provided as Supplementary Data 1–3, respectively.

## Code availability

No custom code is used to generate or process the data described in the manuscript.
