# Supplementary material for: 5G wavelength-division-multiplexing-based bidirectional optical wireless communication system with signal remodulation employing cascaded reflective semiconductor optical amplifiers
Source: Commun Eng. 2024 Jun 22;3:85. doi: 10.1038/s44172-024-00232-1 (PMC11193790; doi:10.1038/s44172-024-00232-1)
Supplement: Supplementary file 1 — Description of Additional Supplementary Files [file 44172_2024_232_MOESM1_ESM.pdf]

Description of Additional Supplementary Files

File name: Supplementary Data 1

Description: Data of Fig. 2(a) for BERs at different received MMW powers over 100-m optical wireless link, at wavelengths of λ1 and λ2 (downlink/uplink).

File name: Supplementary Data 2

Description: Data of Fig. 2(b) for EVMs at different received MMW powers over 100-m optical wireless link, at wavelengths of λ1 and λ2 (downlink/uplink).

File name: Supplementary Data 3

Description: Data of Fig. 5 for the subcarrier EVMs of the 9.1-Gbit/s/28GHz (downlink) and 9.1-Gbit/s/24-GHz (uplink) 16-QAMOFDM signals at different subcarrier indices, in the scenarios of using one RSOA and two RSOAs.
